# Parkinson’s Disease Risk and Hyperhomocysteinemia: The Possible Link

**DOI:** 10.1007/s10571-023-01350-8

**Published:** 2023-04-19

**Authors:** Hayder M. Al-kuraishy, Ali I. Al-Gareeb, Yaser Hosny Ali Elewa, Mahmoud Hosny Zahran, Athanasios Alexiou, Marios Papadakis, Gaber El-Saber Batiha

**Affiliations:** 1 Department of Clinical Pharmacology and Medicine, College of Medicine, Al-Mustansiriya University, Baghdad, Iraq; 2 Department of Histology and Cytology, Faculty of Veterinary Medicine, Zagazig University, Zagazig, Egypt; 3 Faculty of Veterinary medicine , Hokkaido University, Sapporo, Japan; 4 Internal Medicine Department, Faculty of Medicine, Zagazig University, Zagazig, 44519 Egypt; 5 Department of Science and Engineering, Novel Global Community Educational Foundation, Hebersham, NSW 2770 Australia; 6 AFNP Med, 1030 Vienna, Austria; 7 Department of Surgery II, University Hospital Witten-Herdecke, University of Witten-Herdecke, Heusnerstrasse 40, 42283 Wuppertal, Germany; 8 Department of Pharmacology and Therapeutics, Faculty of Veterinary Medicine, Damanhur University, Damanhur, AlBeheira, 22511 Egypt

**Keywords:** Parkinson's disease, Degenerative brain disorders, Hyperhomocysteinemia

## Abstract

Parkinson’s disease (PD) is one of the most common degenerative brain disorders caused by the loss of dopaminergic neurons in the substantia nigra (SN). Lewy bodies and -synuclein accumulation in the SN are hallmarks of the neuropathology of PD. Due to lifestyle changes and prolonged L-dopa administration, patients with PD frequently have vitamin deficiencies, especially folate, vitamin B6, and vitamin B12. These disorders augment circulating levels of Homocysteine with the development of hyperhomocysteinemia, which may contribute to the pathogenesis of PD. Therefore, this review aimed to ascertain if hyperhomocysteinemia may play a part in oxidative and inflammatory signaling pathways that contribute to PD development. Hyperhomocysteinemia is implicated in the pathogenesis of neurodegenerative disorders, including PD. Hyperhomocysteinemia triggers the development and progression of PD by different mechanisms, including oxidative stress, mitochondrial dysfunction, apoptosis, and endothelial dysfunction. Particularly, the progression of PD is linked with high inflammatory changes and systemic inflammatory disorders. Hyperhomocysteinemia induces immune activation and oxidative stress. In turn, activated immune response promotes the development and progression of hyperhomocysteinemia. Therefore, hyperhomocysteinemia-induced immunoinflammatory disorders and abnormal immune response may aggravate abnormal immunoinflammatory in PD, leading to more progression of PD severity. Also, inflammatory signaling pathways like nuclear factor kappa B (NF-κB) and nod-like receptor pyrin 3 (NLRP3) inflammasome and other signaling pathways are intricate in the pathogenesis of PD. In conclusion, hyperhomocysteinemia is involved in the development and progression of PD neuropathology either directly via induction degeneration of dopaminergic neurons or indirectly via activation of inflammatory signaling pathways.

## Introduction

Parkinson's disease (PD) is one of the second most common chronic degenerative brain motor disorders, next to Alzheimer’s disease (AD) (Blauwendraat et al. 2019; Batiha et al. 2022). One percent of people over the age of sixty have PD. Dr. James Parkinson first identified PD in 1817 and described it as a shaking palsy (Kalia and Lang 2015). PD is a progressive disease due to dopaminergic neuron loss in the substantia nigra (SN) with high dopamine deficiency in the basal ganglion (Armstrong and Okun 2020; Al-Kuraishy et al., 2020). PD is characterized by motor and non-motor symptoms. The non-motor symptoms appear before the onset of motor symptoms for many years. Motor signs of PD include rigidity, resting tremors, bradykinesia, and walking difficulty (Lang et al. 2022). Apathy, sadness, anxiety, autonomic disorders, dementia, neuropsychiatric diseases, cognitive dysfunction, and sleep disturbances are the most common non-motor disorders in PD (Yang et al. 2020). PD neuropathology is characterized by the deposition of α-synuclein in the SN, with the formation of Lewy bodies a hallmark of this disease (Church 2021).

Interestingly, the α-synuclein aggregation is not restricted to the SN but affects the entire brain, such as the autonomic nervous system (ANS) (Carapellotti et al. 2020). Furthermore, previous reports documented that the aggregation of α-synuclein is progressive for many years before the development of a symptomatic period (Chen et al. 2019). In fact, the dorsal motor nucleus of the glossopharyngeal and vagus nerves is where -synuclein deposition first begins in the ANS before spreading to other parts of the brain (Norcliffe-Kaufmann 2019). Noticeably, in the prodromal phase, non-motor symptoms, including anosmia, constipation, sleep disorders, and depression, develop before dopaminergic degeneration in the SN (Durcan et al. 2019). Subsequent development of motor symptoms due to dopaminergic degeneration in the SN cognitive dysfunctions is promulgating due to the involvement of the temporal cortex (Kalia 2018).

**Fig. 1:**
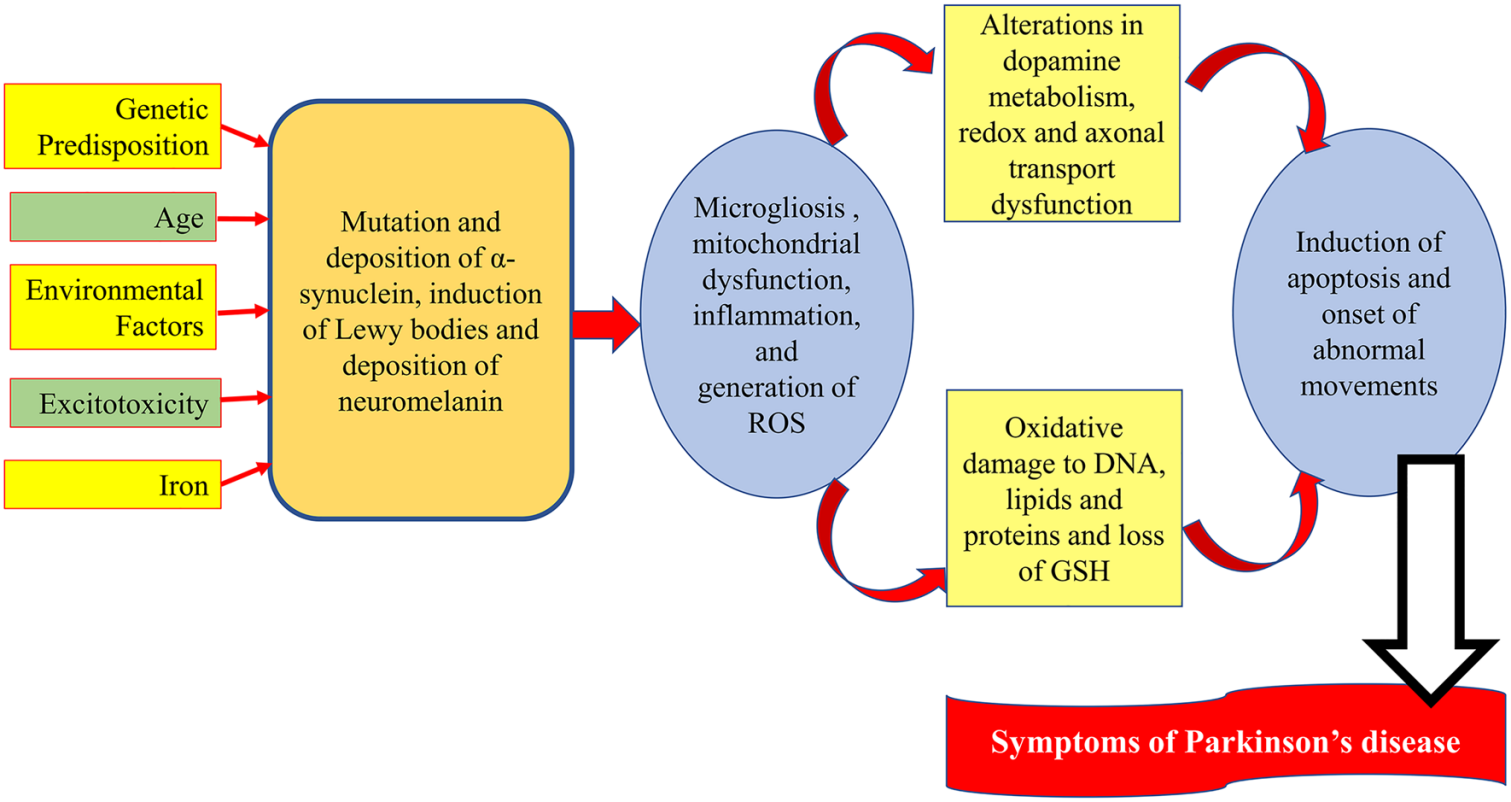
Neuropathology of Parkinson’s disease

Additionally, PD is tied to the development of several inflammatory and oxidative stress illnesses linked to the development of PD neuropathology (Yang et al. 2019). Different factors are involved in the pathogenesis of PD, including old age, genetic and environmental factors causing increasing deposition of α-synuclein and the formation of Lewy bodies (Rai et al. 2020a, Rai et al. 2020b; Rai et al. 2021). In addition to dopaminergic neuronal loss in the SN and the onset of motor symptoms in PD, these changes cause microgliosis, mitochondrial failure, oxidative stress, and inflammation. Therefore, PD neuropathology is complex and related to different factors (Yang et al. 2019; Kalia 2018) [Fig. 1].

Due to lifestyle changes and personality disorders, PD patients are more likely to experience nutritional problems and a lack of specific vitamins, including folate, B6, and B12. Additionally, prolonged L-dopa therapy is linked to folate, B6, and B12 deficiency (de Lau et al. 2006; Christine et al. 2018). These nutritional disorders augment the level of Homocysteine in the blood, leading to hyperhomocysteinemia, which may play a role in the pathogenesis of PD. Therefore, this review aimed to find the potential role of hyperhomocysteinemia in the pathogenesis of PD regarding oxidative and inflammatory signaling pathways.

## Homocysteine Pathway

**Fig. 2:**
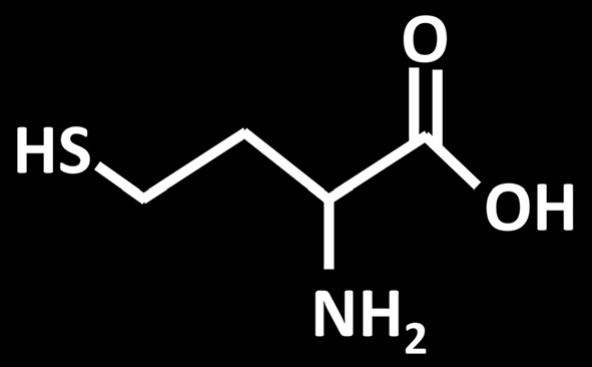
Chemical structure of Homocysteine

Homocysteine is a sulfur and thio-containing amino acid [Fig. 2] produced by methionine demethylation through methionine demethylase, and is involved in the metabolism of methionine and cysteine (Smith and Refsum 2021).

**Fig. 3:**
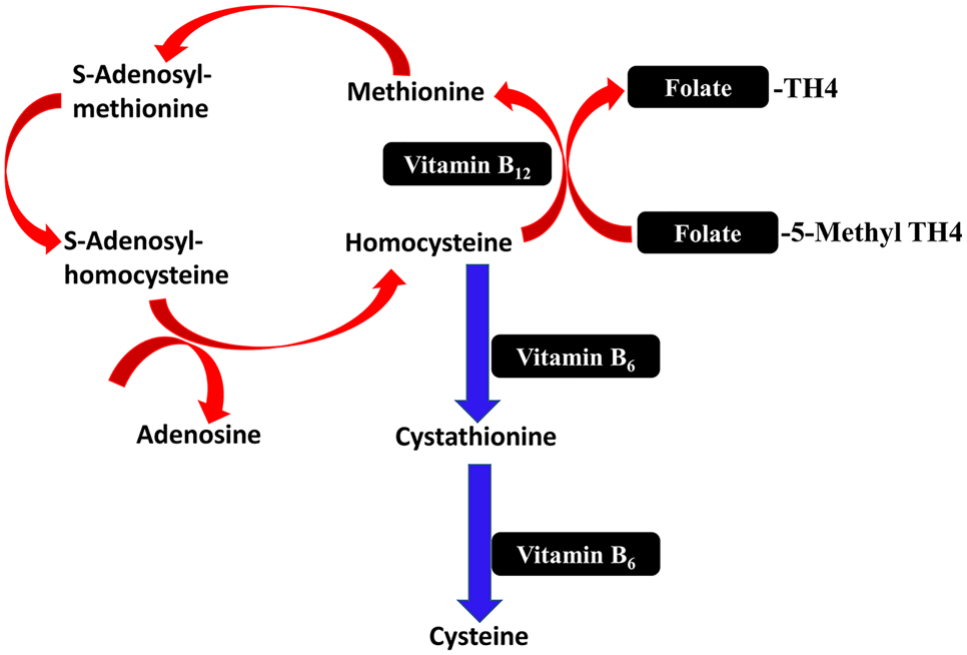
Pathway of Homocysteine: homocysteine, with the assistance of folate and vitamin B12, is converted to methionine and converted to cysteine with the assistance of vitamin B6

Homocysteine is mainly produced from methionine found in the diet; however, it does not contribute to the synthesis of proteins because it is a non-proteinogenic amino acid (Smith and Refsum 2021). About 80% of plasma homocysteine is bound to albumin, though some portions remain free or bound to cystein to form homocysteine-cystein disulfide (Silla et al. 2019). Homocysteine in the body is recycled to form methionine or converted to cysteine with the assistance of vitamins B6, B12, and folate (Silla et al. 2019) [Fig. 3]. Homocysteine can also transform into homocysteine thiolactone in a self-loop reaction, which boosts the generation of reactive oxygen species (ROS) with the development of oxidative stress (Silla et al. 2019; Karolczak and Watala 2021). The average plasma level of Homocysteine is around 10–20 mol/L which is higher in men than in women. Hyperhomocysteinemia is homocysteine plasma levels greater than 15 mol/L, linked to aging and a lack of folate, B6, and B12. Homocysteine levels between 15 and 30 mol/L are recognized as mild hyperhomocysteinemia, between 30 and 100 mol/L as moderate hyperhomocysteinemia, and beyond 100 mol/L as severe hyperhomocysteinemia (Elshahid et al. 2020; Al-Gareeb et al. 2016). Three changes occur to Homocysteine in the plasma: it is remethylated to form methionine, trans-sulfated with serine, and discharged into extracellular fluids (Myles et al. 2008).

The causes of hyperhomocysteinemia may be nutritional deficiencies such as a lack of folate, vitamin B6, or vitamin B12, or they may be hereditary, such as congenital hyperhomocysteinemia caused by a methionine synthase deficiency (Al Mutairi 2020). Hyperhomocysteinemia is thought to be primarily influenced by aging. Age-related increases in plasma homocysteine are previously reported (Xu et al. 2020). Homocysteine levels are positively correlated with age, which may be caused by deficiencies in folate, vitamin B6, and vitamin B12, kidney impairment, and reduced activity of the enzymes involved in the elimination of Homocysteine (Al Mutairi 2020; Xu et al. 2020). Additionally, men may have greater plasma homocysteine levels due to hormonal influences, particularly testosterone. However, even after menopause, homocysteine plasma levels do not vary (Nakhai Pour et al. 2006). Notably, 70% of plasma homocysteine is eliminated by the kidney; thus, renal impairment could potentially cause hyperhomocysteinemia development (Kaplan et al. 2020). Moreover, smoking, alcoholism, and malignancies are associated with hyperhomocysteinemia risk (Kim et al. 2018a; Baszczuk and Kopczyński 2014).

**Fig. 4:**
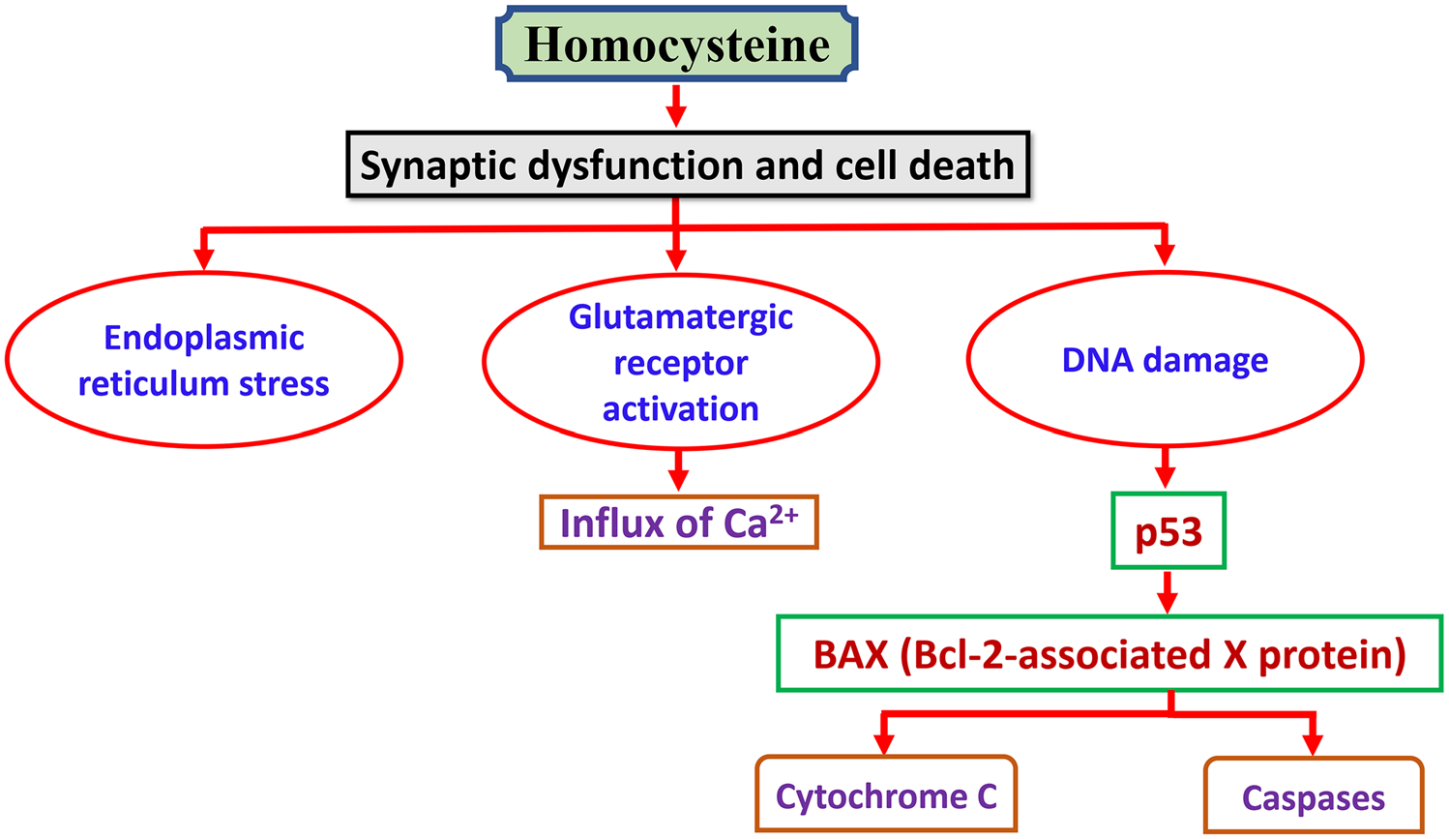
Homocysteine and synaptic dysfunction

It’s interesting to note that hyperhomocysteinemia is linked to the onset of thrombosis, ischemic heart disease, and atherosclerosis (Kravchuk 2012; Al-Kuraishy et al. 2016). It is also believed that hyperhomocysteinemia during pregnancy poses a separate risk for abortion and neural tube defects (Dai et al. 2021). Likewise, Homocysteine leads to synaptic dysfunction by induction of endoplasmic reticulum stress, activation of glutamatergic receptors and DNA damage (Yakovleva et al. 2020) [Fig. 4].

## Hyperhomocysteinemia and Degenerative Brain Diseases

Vascular dementia, AD, PD, and other neurodegenerative illnesses are all linked to hyperhomocysteinemia (Price et al. 2018). Apoptosis, DNA damage, excitotoxicity, and oxidative stress may all play a role in developing hyperhomocysteinemia-induced neurodegenerative disorders (Cordaro et al. 2021). Alteration in the Homocysteine caused by genetic or dietary variables promotes neuronal Ca^+2^, the deposition of tau and amyloid beta (Aβ), and the induction of neuronal cell deaths and apoptosis (Cordaro et al. 2021). The risk of stroke and the onset of vascular dementia had been linked to hyperhomocysteinemia and related microangiopathy (Kevere et al. 2012). Furthermore, hyperhomocysteinemia increases the risk of an ischemic stroke by inhibiting the endogenous anticoagulant system and increasing thrombin production (Faverzani et al. 2017). Also, hyperhomocysteinemia triggers platelet activation by increasing lipid peroxidation and oxidative stress effects of hyperhomocysteinemia on the platelet-driven contraction of blood clots (Litvinov et al. 2021). A previous study illustrated that hyperhomocysteinemia increased ischemic risk in children ( Komitopoulou et al. 2006). A case–control study that included 45 patients with ischemic stroke and 234 healthy controls showed higher homocysteine serum levels and was correlated with the risk of ischemic stroke (Komitopoulou et al. 2006). A study comparing 152 healthy controls to 161 patients with ischemic stroke found that hyperhomocysteinemia is an independent risk factor for developing ischemic stroke (Parnetti et al. 2004). These findings indicated that hyperhomocysteinemia is implicated in the development of ischemic stroke and the progression of vascular dementia.

Moreover, hyperhomocysteinemia increases the incidence of multiple sclerosis through macrophage activation and induction of immune deregulations (Teunissen et al. 2008; Ramsaransing et al. 2006). Hyperhomocysteinemia is correlated with the progression of multiple sclerosis due to a defect in the methylation of myelin basic protein with subsequent degeneration of myelin sheath (Ramsaransing et al. 2006; Teunissen et al. 2008). It has been shown that hyperhomocysteinemia induces endothelial dysfunction, impairment of blood brain barrier (BBB) and thrombosis with subsequent translocation of leukocytes and immune cells into CNS (Dubchenko et al. 2020). Besides, hyperhomocysteinemia contributes to progressive neuronal injury and apoptosis (Dubchenko et al. 2020). Therefore, hyperhomocysteinemia directly damages neuronal sheath or indirectly through the induction of abnormal immune response (Mititelu et al. 2021). Additionally, multiple sclerosis’s clinical progression and cognitive impairment are both associated with hyperhomocysteinemia (Teunissen et al. 2008).

Markedly, hyperhomocysteinemia is considered an independent risk factor for AD (Nazef et al. 2014; Alsubaie et al. 2022; Al-Kuraishy et al. 2022a). A case–control study involving 41 AD patients and 46 healthy controls showed that hyperhomocysteinemia correlates with cognitive impairment and AD risk due to induction of cortical atrophy and reduced hippocampal activity (Nazef et al. 2014; Al-Kuraishy et al. 2022b). Of note, hyperhomocysteinemia decreases learning and memory by distorting synaptic transmission and synaptic plasticity in rats (An and Zhang 2013). Therefore, plasma homocysteine level is regarded as a biomarker evaluating the development of AD and other types of dementia (Seshadri et al. 2002). The underlying mechanism linking hyperhomocysteinemia and dementia is the development of endothelial function and impairment of cerebral blood flow with subsequent oxidative stress-induced neuronal injury (Kovalska et al. 2018, 2019; Rehman et al. 2020). Moreover, hyperhomocysteinemia inhibits the inhibitory neurotransmitter gamma-aminobutyric acid (GABA), leading to excitotoxicity and BBB disruption (Tyagi et al. 2007). Remarkably, hyperhomocysteinemia promotes Aβ formation and increases neurons' sensitivity to the toxic effects of Aβ in experimental studies (Zhuo et al. 2011; Chung et al. 2016). Thus, hyperhomocysteinemia induces and exacerbates AD neuropathology via Aβ alone or through interaction with fibrinogen (Chung et al. 2016).

**Fig. 5:**
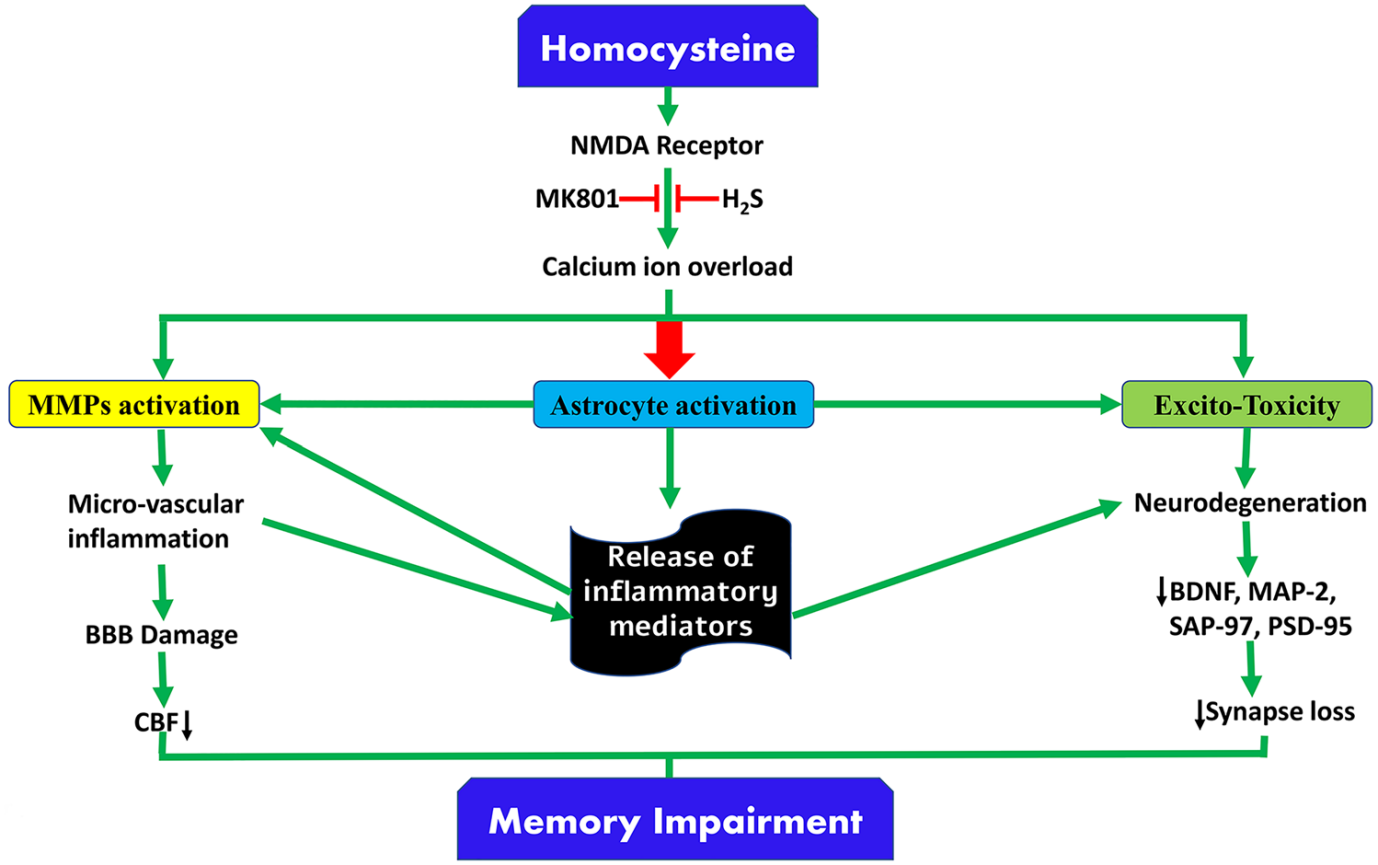
Role of Homocysteine in neurodegeneration and cognitive impairment: homocysteine, through activation of N-Methyl-D-Aspartate (NMDA) receptors, induces Ca^+2^ leading to excitotoxicity, astrocyte activation with release inflammatory mediators and activation of matrix metalloproteinase (MMP) with subsequent BBB injury and microvascular inflammation. These changes cause a reduction of cerebral blood flow (CBF) and synaptic dysfunction with the development of neurodegeneration and cognitive impairment. In addition, neurodegeneration induces a reduction in the expression of brain-derived neurotrophic factor (BDNF), microtubule-associated protein 2 (MAP-2), synapse associate protein 97 (SAP-97), postsynaptic density protein 95 (PSD-95) with the development of synaptic loss

Homocysteine, through activation of NMDA receptors, induces Ca^+2^, leading to excitotoxicity, astrocyte activation with release inflammatory mediators, and matrix metalloproteinase (MMP) activation with subsequent BBB injury and microvascular inflammation (Kamat et al. 2016). In addition, these changes cause a reduction of cerebral blood flow (CBF) and synaptic dysfunction with the development of neurodegeneration and cognitive impairment (Kamat et al. 2016)[Fig. 5].

Taken together, hyperhomocysteinemia is correlated with development and progression of various types of degenerative brain diseases by inducing neuronal oxidative stress and DNA damage.

## Hyperhomocysteinemia and PD

It has been shown that hyperhomocysteinemia is an independent risk factor for the development of PD (Sampedro et al., 2022). A case–control study showed that plasma homocysteine level was higher in PD patients than in healthy controls (Kuhn et al. 1998). A recent cross-sectional study comprising 99 PD patients, 34 with minor hallucinations and 65 without minor hallucinations, revealed that plasma homocysteine level was higher in PD patients with minor hallucinations (Zhong et al. 2022). This study suggests that plasma homocysteine level is correlated with motor and non-motor manifestations like psychiatric disorders in PD. As well, plasma homocysteine level predicts the clinical outcomes in PD patients (Zhong et al. 2022). It has been noted that patients with PD and AD have higher levels of total CSF homocysteine but not free Homocysteine (Isobe et al. 2005). Therefore, the total homocysteine level in CSF may serve as a diagnostic biomarker for both PD and AD. The use of L-dopa may be responsible for an increase in CSF total Homocysteine in PD patients (Isobe et al. 2010).

Moreover, hyperhomocysteinemia leads to memory dysfunction and reduced verbal fluency commonly observed in PD patients due to the development of oxidative stress in the neocortex (Hara et al. 2016).

Muller et al. observed that initiating L-dopa therapy in PD patients induces the development of hyperhomocysteinemia (Müller and Kuhn 2009). This may explain the propagation of neuropsychiatric disorders and atherosclerotic complications in PD patients. Notably, L-dopa impairs homocysteine metabolism and elimination, leading to hyperhomocysteinemia and associated disorders, as confirmed by a cohort study (Müller and Kuhn 2009). However, a previous study illustrated a modest increase in plasma homocysteine levels following the initiation of L-dopa therapy in PD patients (O’Suilleabhain et al. 2004). Furthermore, a prospective study involving PD patients on L-dopa therapy compared to other treatments showed that L-dopa therapy in PD patients was associated with a modest increase in plasma homocysteine level with a significant reduction in B12 serum level (O’Suilleabhain et al. 2004). Therefore, L-dopa therapy and vitamin deficiency increase the risk of PD severity. Thus, folate and 12 supplementations improve hallucination, sleep disorders, and motor disorders in PD patients by ameliorating hyperhomocysteinemia-induced oxidative stress and inflammatory disorders (Srivastav et al. 2015; Haghdoost-Yazdi et al. 2012). In addition, hyperhomocysteinemia leads to a differential gender-specific effect on cognitive and motor severity in PD patients (Bakeberg et al. 2019). A case–control study revealed that hyperhomocysteinemia led to more detrimental effect in men compared to women with PD by unknown mechanism (Bakeberg et al. 2019).

**Fig. 6:**
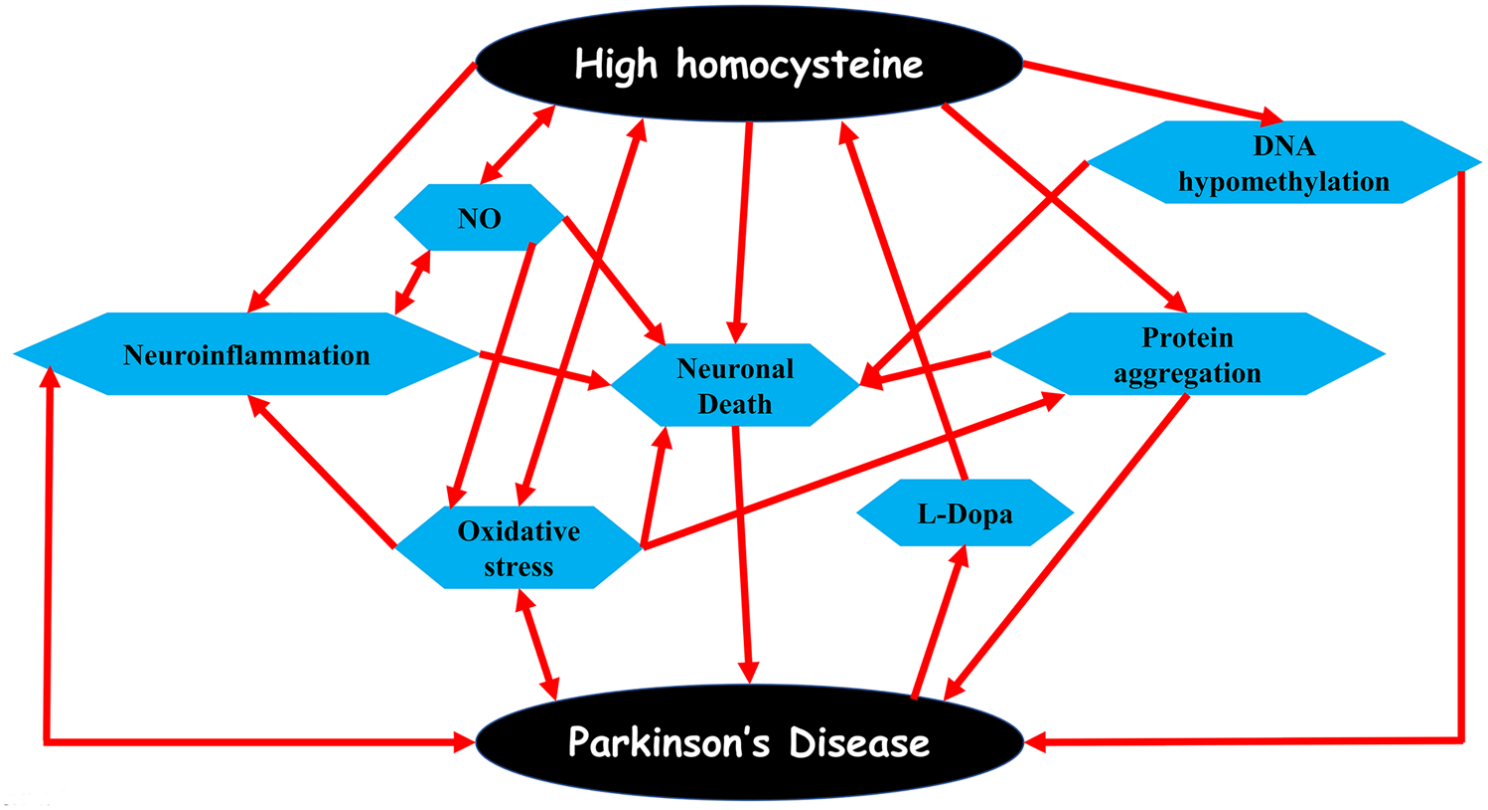
The link between high Homocysteine and the development of PD

The complex interaction between hyperhomocysteinemia and PD induces different pathological changes, including DNA hypomethylation, neuroinflammation, oxidative stress, and neuronal cell deaths (Doherty 2013). These verdicts proposed a potential link between hyperhomocysteinemia and the development/progression of PD [Fig. 6].

## Hyperhomocysteinemia and Degeneration of Dopaminergic Neurons

Homocysteine acts directly as an NMDA receptor agonist or indirectly by inhibiting GABA leading to excitotoxicity and neuronal Ca^+2^ overloads with the acceleration of protein misfolding and Aβ aggregation (Hassin-Baer et al. 2006). It has been shown that glutamate-induced excitotoxicity is linked to the development and progression of PD (Iovino et al. 2020). Accumulating extra-synaptic glutamate due to the over-activation of microglia promotes aberrant synaptic signaling in PD and other neurodegenerative brain diseases (Iovino et al. 2020). A computational study demonstrated that glutamate-induced excitotoxicity is correlated with the loss of dopaminergic neurons in PD (Muddapu et al. 2019). In this bargain, a case–control study involving 110 PD patients compared to 90 healthy controls observed that serum glutamate level was higher in PD patients (Mironova et al. 2018).

Together, GABA signaling is highly disturbed in PD and associated with developing motor and non-motor symptoms (van Nuland et al. 2020). A case–control study illustrated that cortical GABA benefits PD patients by reducing motor symptoms (van Nuland et al. 2020). GABA-ergic dysfunction also contributes to the development of non-motor symptoms in PD (Murueta-Goyena et al. 2019). Cognitive dysfunction and motor and non-motor symptoms in PD may be due to disturbance of GABA and adenosine signaling (Zhao et al., 2021). It has been shown that arbutin in plants can improve various motor functions, including posture, movement, and rigidity, in MPTP-treated mice. Arbutin exhibited potent antioxidant and anti-inflammatory activities and could restore neurotransmitter levels like dopamine and GABA in the striatum and protect neurons against degeneration through inhibition of adenosine signaling (Zhao et al., 2021). GABAergic neurons play a critical role in the modulation of the activity of the thalamocortical motor circuit in PD (van Nuland et al. 2020). A study involved 60 PD patients with dopamine-resistant tremor (*n* = 17), dopamine-responsive tremor (*n* = 23), or no tremor (*n* = 20), and healthy controls (*n* = 22) showed that GABA level was unchanged in PD patients compared to the controls. Though, motor cortex GABA level was inversely correlated with disease severity. Therefore, cortical GABA has a beneficial rather than a detrimental role in PD, and GABA reduction may donate to increased motor symptom expression (van Nuland et al. 2020). Moreover, dysregulation of GABAergic signaling is linked with the development of non-motor symptoms, including sleep disorders in PD (Murueta-Goyena et al. 2019). Thus, hyperhomocysteinemia may aggravate PD through augmentation of glutamate-induced excitotoxicity and attenuation of the protective effect of GABA.

Homocysteine also triggers ROS generation and reduces the generation of nitric oxide (NO) with activation of inflammatory series leading to progressive neuronal loss (Hassin-Baer et al. 2006). A cohort study noted that hyperhomocysteinemia is associated with the progression of systemic oxidative stress in post-menopausal women (Bourgonje et al. 2020). Zhang et al. (Zhang et al. 2020) showed that hyperhomocysteinemia induces ferroptosis and oxidative stress by enhancing the methylation of glutathione peroxidase. Bhattacharjee and Borah found that mitochondrial dysfunction and the development of oxidative stress could be the potential mechanisms for homocysteine-induced degeneration of dopaminergic neurons in the SN in the rat model of PD (Bhattacharjee and Borah 2016). Of note, oxidative stress and reduction of glutathione peroxidase activity are linked with the degeneration of dopaminergic neurons in PD (Aborode et al. 2022). A systematic review and meta-analysis involving 80 studies of 7212 PD patients and 6037 healthy subjects revealed that PD is linked with higher oxidative stress biomarkers (Wei et al. 2018). Herein, hyperhomocysteinemia may aggravate PD through the induction of oxidative stress; when the plasma homocysteine level progressively rises, it is associated with an increase in both the motor and non-motor symptoms of PD.

A previous experimental study conducted by Lee et al. found that Homocysteine reduced tyrosine hydroxylase (TH) activity in the SN with a significant reduction in dopamine turnover in mice and rats. In addition, L-dopa treatment in PD augments the toxic effect of Homocysteine on the dopaminergic neurons in the SN (Lee et al. 2005). In vitro studies demonstrated that TH-positive neurons were highly susceptible to the toxic effect of Homocysteine (Heider et al. 2004). In addition, increased intracellular dopamine enhances the toxic effect of Homocysteine (Heider et al. 2004). Likewise, hyperhomocysteinemia depletes neuronal ATP and enhances the sensitivity of dopaminergic neurons to the toxic effect of rotenone in rats (Duan et al. 2002). In this state, L-dopa treatment in the MPTP model of PD augments homocysteine neurotoxicity without a reduction in the number of dopaminergic neurons (Bhattacharjee et al. 2016). Indeed, human TH activity is related to PD neuropathology and other neurodegenerative brain diseases (Nagatsu et al. 2019). The reduction of TH activity could be secondary to the degeneration of dopaminergic neurons in PD. Thus, TH activity deficiency may not contribute to PD neuropathology (Nagatsu et al. 2019). Also, the accumulation of α-synuclein increases the depletion of TH (Kawahata and Fukunaga 2020). Thus, the depletion of TH in the SN is not the primary event in the pathogenesis of PD. Therefore, hyperhomocysteinemia-induced depletion of TH may not involve in the development but only in the progression of PD through the degeneration of dopaminergic neurons with secondary deficiency of TH in PD (Kawahata and Fukunaga 2020; Nagatsu et al. 2019).

The direct toxic effect of Homocysteine on the dopaminergic neurons in the SN could be the possible mechanism in the development and progression of PD. It has been reported that Homocysteine augments the CNS to the toxic methylation process. Homocysteine inhibits S-adenosyl-homocysteine (SAH) metabolism causing increasing of SAH with induction of apoptosis and neuronal injury with the development of cognitive impairment (Lin et al. 2008). SAH level was reported to be higher in PD patients than in healthy controls (Kennedy et al. 2004). A study involving 87 PD patients revealed that methylation biomarkers, including SAH, were increased (Obeid et al. 2009). SAH level was correlated with methyltransferase inhibition and cognitive impairment in PD and AD patients (Kennedy et al. 2004). Of interest, direct exposure of dorsal hippocampus to the effect of Homocysteine does not cause direct neurotoxicity, though co-administration of Homocysteine with glutamate agonists like kainic acid induces more neurotoxicity (Müller et al. 2001; Kruman et al. 2000). These findings suggest that Homocysteine does not cause direct neurotoxicity but enhance the sensitivity of dopaminergic neurons to the environmental toxins.

Furthermore, homocysteine-induced apoptosis is mainly mediated by induction DNA injury and energy depletion (Fan et al. 2019). Homocysteine reduces ATP production and cellular glucose consumption by inhibiting cytochrome C oxidase leading to more cellular injury (Zhai et al. 2019). Plasma mitochondrial and nuclear DNA levels were increased in PD patients correlated with autonomic dysfunction (Chen et al. 2017). These biomarkers served as mediators of autonomic dysfunction, like poor baroreflex reaction and sensitivity in PD patients (Chen et al. 2017).

Reduction of homocysteine conversion to methionine due to deficiency of vitamin B12 and folic acid triggers DNA injury (Koklesova et al. 2021). These changes provoke neuronal apoptosis by inhibiting mitochondrial dysfunction and developing oxidative stress (Koklesova et al. 2021). Homocysteine-induced mitochondrial dysfunction is mediated by the activation of caspase activity and distortion of mitochondrial trans-membrane potential leading to Ca^+2^ overload and apoptosis (Wang et al. 2018). It has been shown that apoptosis plays a crucial role in PD neuropathology. Apoptosis is initiated by caspase-9 and caspase-8, leading to DNA cleavage and fragmentation (Babalghith et al., 2022). Pro-apoptotic factors like Bax promote caspase-mediated dopaminergic neuronal injury and the development of PD (Erekat 2018). Therefore, homocysteine-induced apoptosis and DNA damage could be potential mechanisms for the development and progression of PD in patients with hyperhomocysteinemia. Together, oxidative stress, mitochondrial dysfunction, and apoptosis are interrelated in the induction of dopaminergic neurodegeneration and development of PD (Javed et al. 2020).

Moreover, hyperhomocysteinemia is the leading cause of endothelial dysfunction and the development of atherosclerosis by direct injury of endothelial cells (Esse et al. 2019; Al-kuraishy et al. 2022c). Homocysteine interacts with various molecules produced from endothelial cells, including thrombomodulin and Von-Willebrand factor leading to disturbance of the endothelial coagulant/anticoagulant axis with the development of thrombosis (Nilsson et al. 2005). Besides, Homocysteine inhibits endothelial glutathione peroxidase by reducing endogenous antioxidant capacity (Wu et al. 2019). Further, Homocysteine activates vascular smooth muscle hyperplasia with further aggravation of endothelial dysfunction (Balint et al. 2020). Endothelial dysfunction is associated with the progression of nigrostriatal injury and the development of PD (Cahill-Smith and Li 2014). Interestingly, a cohort study revealed that L-dopa therapy in PD patients increases the risk of homocysteine-induced endothelial dysfunction with the progression of PD neuropathology (Yoon et al. 2014).

**Fig. 7:**
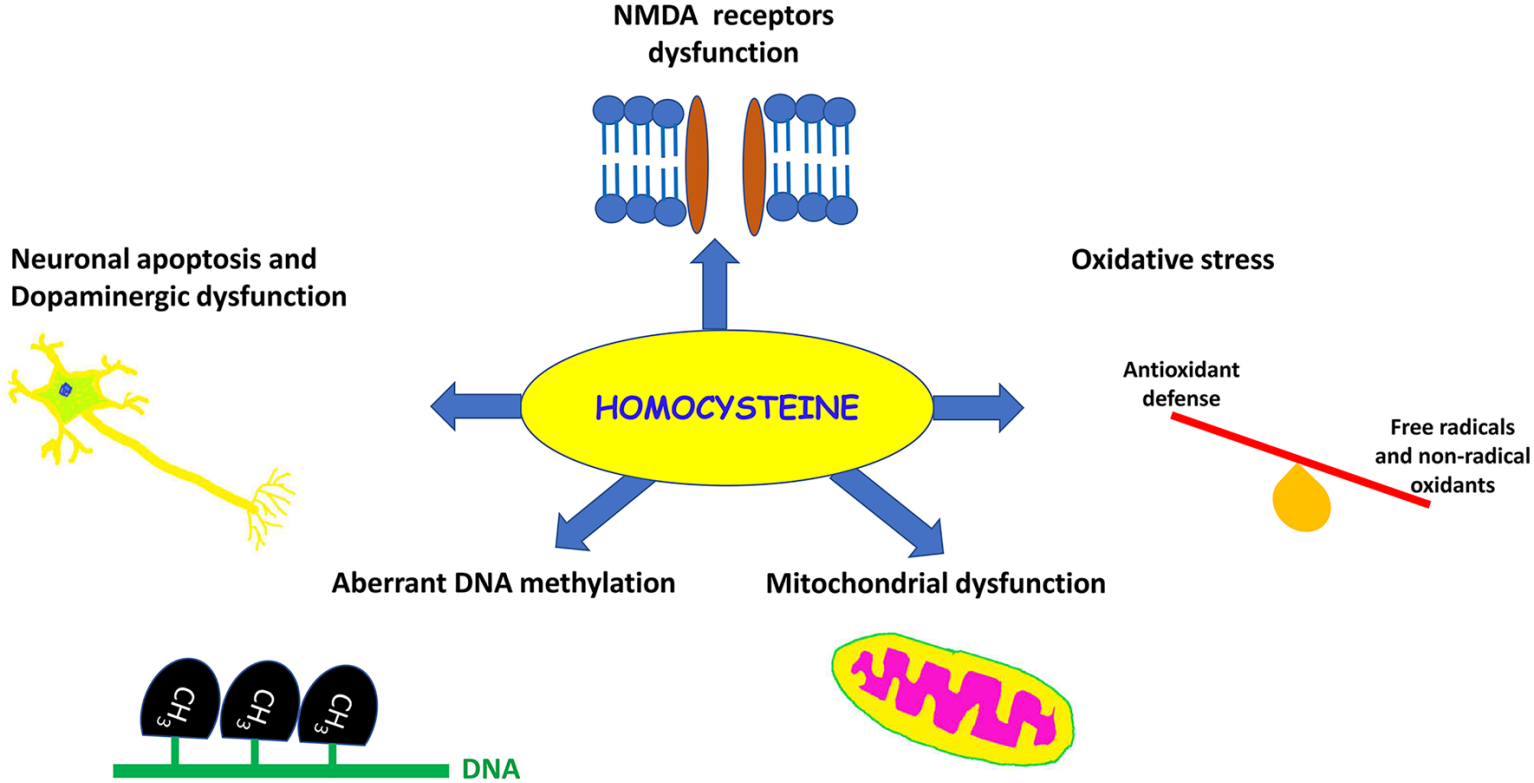
Mechanism of homocysteine role in Parkinson’s disease

Taken together, hyperhomocysteinemia triggers the development and progression of PD by different mechanisms, including oxidative stress, mitochondrial dysfunction, apoptosis, and endothelial dysfunction [Fig. 7].

## Hyperhomocysteinemia and Immunoinflammatory Response in PD

It has been shown that the advancement of PD is linked with high inflammatory changes and systemic inflammatory disorders (Lin et al. 2016). Notably, pro-inflammatory cytokines are increased in the peripheral blood cells of PD patients (Koziorowski et al. 2012). Furthermore, a prospective study that included 60 PD patients compared to 24 healthy controls exposed that pro-inflammatory cytokines are increased in PD patients (Koziorowski et al. 2012). These judgments designated that higher inflammatory changes may intensify the development of PD. Besides, Homocysteine induces transcription of inflammatory mediators in monocytes (Meng et al. 2013). Thus, Homocysteine is regarded as a pro-inflammatory amino acid that induces the expression of different transcription factors and the release of pro-inflammatory cytokines (Meng et al. 2013).

Furthermore, an experimental study demonstrated that chronic hyperhomocysteinemia augments inflammatory biomarkers in rat hippocampus (da Cunha et al. 2012). In contrast, pro-inflammatory cytokine levels are not correlated with homocysteine plasma levels in AD patients (Veryard et al. 2013). Thus, mild hyperhomocysteinemia in AD may not be associated with inflammatory reactions. Notoriously, hyperhomocysteinemia-induced brain injury and disruption of BBB are mediated by the release of pro-inflammatory cytokine and immunomodulatory dysfunction to protect the injured brain (Zhang et al. 2019).

Noteworthy, the dysfunction of the immune system with genetic susceptibility impairs humoral and cellular immune response in PD (Tan et al. 2020). Dysregulation of the immune system and abnormal innate/adaptive immune response are concerned with developing degenerative brain diseases including PD (Tan et al. 2020). In PD, an immune response is slanted with accumulative risk for the development of the autoimmune response. Consequently, peripheral inflammatory biomarkers may be augmented and connected with motor severity in PD patients (Kim et al. 2018b). A case–control study involving 58 PD patients compared to 20 healthy controls showed that IL-1β, TNF-α, IL-6, CRP, and IL-12 are increased in PD patients compared to healthy controls (Kim et al. 2018b). There was no positive correlation between levels of inflammatory biomarkers and non-motor symptoms in PD patients (Kim et al. 2018b). Chen (2005) showed an unusual alteration in inflammatory cytokines in the CSF of patients with degenerative brain diseases, including PD. Furthermore, abnormal immune response and microglia hyper-activation are connected with the degeneration of dopaminergic neurons in the SN (Miller et al. 2009). These findings indicated that the progression of PD is highly correlated with the severity of peripheral inflammatory disorders.

On the other hand, moderate hyperhomocysteinemia induces immune activation through ROS and oxidative stress (Schroecksnadel et al. 2004). In turn, an activated immune response promotes the development and progression of hyperhomocysteinemia (Schroecksnadel et al. 2004). Lazzerini and colleagues found that hyperhomocysteinemia is intricate in developing inflammation and autoimmunity (Lazzerini et al. 2007). Similarly, immunoinflammatory reactions contribute to the development of hyperhomocysteinemia, and in turn, high Homocysteine acts as an immunostimulant and pro-inflammatory molecule, increasing abnormal immune response (Lazzerini et al. 2007). Notably, Homocysteine can react and modify specific proteins, resulting in neo-antigens development and autoimmunity development (Lazzerini et al. 2007). Further, hyperhomocysteinemia triggers immune imbalance and inflammation during acute brain injury (Zhang et al. 2021).

Therefore, hyperhomocysteinemia-induced immunoinflammatory disorders and abnormal immune response may aggravate abnormal immunoinflammatory in PD, leading to more progression of PD severity.

## Hyperhomocysteinemia and Inflammatory Signaling Pathways in PD

Inflammatory signaling pathways like nuclear factor kappa B (NF-κB) and nod-like receptor pyrin 3 (NLRP3) inflammasome as well as other signaling pathways, are intricate in the pathogenesis of PD (Miller et al. 2009; Batiha et al. 2022b).

### NF-κB

NF-κB is a DNA-binding protein prerequisite for transcription pro-inflammatory cytokines and chemokines. Although NF-κB is under the control of extracellular stimuli, it is inhibited by an inhibitor of κB (IκB) which sequester NF-κB in the cytosol and prevent its localization (Al-Kuraishy et al. Kuraishy et al. 2021; Al-Kuraishy et al. 2023). However, cytokines inhibit IκB with subsequent activation of NF-κB and propagation of inflammatory disorders (Ladner et al. 2003; Chen 2005).

NF-κB is also intricate in the pathogenesis of PD via induction of inflammation-mediated degeneration of dopaminergic neurons in the SN (Singh et al. 2020). Immune dysregulation by aging promotes the activation of NF-κB with subsequent neuronal injury and neuroinflammation with the development of PD (Singh et al. 2020). Results from postmortem studies advocate the role of NF-κB in the degeneration of dopaminergic neurons in the SN. Activation of NF-κB with induction of neuronal apoptosis was established in PD patients compared to the controls (Hunot et al. 1997). Ghosh et al. (Ghosh et al. 2007) exemplified that selective inhibition of NF-κB prevents the degeneration of dopaminergic neurons in the SN in the mouse model of PD.

Similarly, targeting the NF-κB pathway in murine and mouse PD models may prevent PD progression (Flood et al. 2011). Notably, different drugs and herbals like pioglitazone, salmeterol, and curcumin hinder the degeneration of dopaminergic neurons in the SN by inhibiting NF-κB which is involved in the progression of neuroinflammation and injury of dopaminergic neurons (Flood et al. 2011; Al-kuraishy et al. 2020). Furthermore, a recent finding demonstrated that α-synuclein released from injured dopaminergic neurons triggers activation of NF-κB and release of pro-inflammatory cytokines with further aggravation of dopaminergic neurons in a positive-loop fashion (Dolatshahi et al. 2021). These findings proposed that NF-κB could be a therapeutic target in the management of PD.

Curiously, the Aβ_1-42_ level in the CSF is reduced and not correlated with motor dysfunction in PD patients compared to the controls (Buddhala et al. 2015). Shi et al. (Shi et al. 2011) exposed that the Aβ_1-42_ level in the CSF is augmented and interrelated with the severity of PD. Nonetheless, Aβ_1-42_ inhibits BBB P-glycoprotein through induction of NF-κB with further reduction in clearance of Aβ_1-42_ (Park et al. 2014). Therefore, NF-κB not only induces dopaminergic neurons in the SN but also increases the PD severity through accumulation of Aβ_1-42_ and α-synuclein.

Furthermore, hyperhomocysteinemia triggers the activation of NF-κB, causing releasing of pro-inflammatory cytokines (Ferlazzo et al. 2008). This could be a putative mechanism for the induction of neurotoxicity by hyperhomocysteinemia. In vitro study demonstrated that the addition of NF-κB inhibitor abolishes hyperhomocysteinemia-induced neuronal apoptosis (Ferlazzo et al. 2008). Similarly, through the inhibition development of hyperhomocysteinemia, folic acid mitigates palmitate-induced inflammation and NF-κB activation in HepG2 cells (Bagherieh et al. 2021). Captopril also attenuates homocysteine-induced inflammation via the NF-κB signaling pathway in human aorta endothelial cells (Hu et al. 2019). An experimental study demonstrated that hyperhomocysteinemia triggers oxidative stress and inflammation via activating the NF-κB signaling pathway in the cerebellum and striatum of rodents (Dos Santos et al. 2021). Thus, hyperhomocysteinemia-induced NF-κB activation could be a possible mechanism for arterial injury in patients with premature coronary artery diseases (Liu et al. 2022).

These verdicts indicated that hyperhomocysteinemia augments the inflammatory burden in PD patients through activation of the NF-κB signaling pathway involved in the development and progression of PD.

### NLRP3 Inflammasome

NLRP3 inflammasome is the nucleotide-binding domain, and the leucine-rich repeat-containing family and pyrin family can form a multiprotein complex. The main function of NLRP3 inflammasome is an activation of caspase-1, maturation of IL-1β and IL-18 (He et al. 2016). NLRP3 inflammasome is activated by different stimuli including alternative and non-canonical pathways (He et al. 2016). NLRP3 inflammasome is activated by NF-κB and sphingosine-1 phosphate (Paik et al. 2021).

NLRP3 inflammasome is intricate in the pathogenesis of PD (Haque et al. 2020). NLRP3 inflammasome induces the release of pro-inflammatory cytokines and the development of neuroinflammation and degeneration of dopaminergic neurons by induction of pyroptosis (Haque et al. 2020; Wang et al. 2019). The accumulation of the α-synuclein also triggers the microglia's activation with the subsequent expression of NLRP3 inflammasome in the SN (Haque et al. 2020). Furthermore, systemic activation of NLRP3 inflammasome promotes the accumulation of α-synuclein and degeneration of dopaminergic neurons in the SN (Fan et al. 2020). A case–control study that included 67 PD patients compared to 24 healthy controls showed that plasma levels of α-synuclein, NLRP3 inflammasome, caspase-1, and IL-1β increased in PD patients compared to healthy patients controls (Fan et al. 2020).

Consequently, α-synuclein, NLRP3 inflammasome, and IL-1β plasma could be biomarkers to monitor PD severity and progression. Different studies showed that higher levels of pro-inflammatory cytokines in the CSF and plasma support the interaction between the brain and the immune system with the development of neuroinflammation and degeneration of dopaminergic neurons in PD (Jiang and Dickson 2018; Qiao et al. 2018). IL-1β plasma level a main component of NLRP3 inflammasome is augmented in PD patients (Boxberger et al. 2019). These observations proposed that systemic inflammation via induction of neuroinflammation may lead to the degeneration of dopaminergic neurons and the development of PD. Additionally, increased α-synuclein plasma level, a major constituent of Lewy bodies, had been reported to be increased in PD patients compared to the healthy controls (Bougea et al. 2019). In turn, α-synuclein can trigger NLRP3 inflammasome with subsequent release of IL-1β with the development of systemic inflammation and neuroinflammation (Codolo et al. 2013).

Moreover, hyperhomocysteinemia induces inflammation by activating NLRP3 inflammasome in ApoE-deficient mice (Wang et al. 2017). The underlying mechanism for hyperhomocysteinemia-induced NLRP3 inflammasome activation is through the generation of ROS (Wang et al. 2017). NLRP3 inflammasome activation is also activated by cholesterol and oxidized low-density lipoprotein (oxLDL) (Duewell et al. 2010). Yang et al. (Yang et al. 2005) observed that hyperhomocysteinemia promotes cholesterol levels and atherosclerosis. Also, hyperhomocysteinemia triggers lipid accumulation and cholesterol biosynthesis by activating rat transcription factors (Woo et al. 2005). Also, hyperhomocysteinemia increases the production of oxLDL in atherosclerotic patients (Seo et al. 2010; Al-kuraishy et al. 2019). Recently, hyperhomocysteinemia has been correlated with high oxLDL levels (Ridker et al. 2022). Thus, hyperhomocysteinemia-induced NLRP3 inflammasome activation is mainly through ROS generation, cholesterol biosynthesis, and generation of oxLDL. Therefore, hyperhomocysteinemia may directly affect PD neuropathology or indirectly through activation of NLRP3 inflammasome.

Taken together, hyperhomocysteinemia through activation of NF-κB and NLRP3 inflammasome signaling pathways may augment PD neuropathology and associated neuroinflammation.

## Conclusion

PD is one of the most prevalent chronic degenerative brain motor disorders. Because of the loss of dopaminergic neurons in the SN and severe dopamine deficiency in the basal ganglia, PD is considered a progressive disease. Lewy bodies formation and α-synuclein deposition in the SN are hallmarks of the neuropathology of PD. However, the accumulation of α-synuclein is not restricted to the SN but affects the entire brain including ANS. PD patients are subjected to nutritional disorders and deficiency of certain vitamins like folate, vitamin B6 and B12. Additionally, prolonged L-dopa therapy is linked to folate, vitamin B6 and B12 deficiency. These disorders augment the circulating level of Homocysteine with the development of hyperhomocysteinemia, which may involve the pathogenesis of PD. Hyperhomocysteinemia is implicated in the pathogenesis of vascular dementia, AD, PD, and other neurodegenerative disorders. Apoptosis, DNA damage, excitotoxicity, and oxidative stress may all play a role in developing neurodegenerative diseases brought on by hyperhomocysteinemia.

Moreover, hyperhomocysteinemia is a separate risk factor for the onset of PD. In addition, the initiation of L-dopa therapy in PD patients causes the development of hyperhomocysteinemia, which may help to explain why neuropsychiatric diseases continue to spread in PD patients. As a result, combining L-dopa medication with a vitamin deficiency raises the risk of developing severe PD. Through various processes, including oxidative stress, mitochondrial malfunction, apoptosis, and endothelial dysfunction, hyperhomocysteinemia triggers the occurrence and progression of PD. Notably, systemic inflammatory disorders are associated with the progression of PD. The immune system is activated by hyperhomocysteinemia via ROS and oxidative stress. Hyperhomocysteinemia then develops and progresses due to active immunological response. Therefore, abnormal immune response and immunoinflammatory diseases brought on by hyperhomocysteinemia may exacerbate abnormal immunoinflammatory in PD.

Furthermore, the pathophysiology of PD involves numerous signaling pathways, including inflammatory signaling pathways like NF-B and NLRP3 inflammasome. Hyperhomocysteinemia may worsen PD neuropathology and related neuroinflammation by activating the NF-B and NLRP3 inflammasome signaling pathways. Together, our findings suggest that hyperhomocysteinemia has a role in the onset, development, and progression of PD neuropathology, directly inducing dopaminergic neuron degeneration or indirectly activating inflammatory signaling pathways. Therefore, preclinical and clinical studies are warranted in this regard.

## Data Availability

Not applicable.

## Abbreviations

AD: Alzheimer’s disease
ANS: Autonomic nervous system
BBB: Blood brain barrier
CNS: Central nervous system
GABA: Gamma aminobutyric acid
NF-κB: Nuclear factor kappa B
NLRP3: Nod-like receptor pyrin 3
PD: Parkinson’s disease
ROS: Reactive oxygen species
SAH: S-adenosyl-homocysteine
SN: Substantia nigra
